# High-sensitivity troponin T as a marker to predict cardiotoxicity in breast cancer patients with adjuvant trastuzumab therapy

**DOI:** 10.1186/2193-1801-3-620

**Published:** 2014-10-20

**Authors:** Kenichi Katsurada, Masaru Ichida, Masako Sakuragi, Megumi Takehara, Yasuo Hozumi, Kazuomi Kario

**Affiliations:** Department of Cardiology, Jichi Medical University School of Medicine, 3311-1 Yakushiji, Shimotsuke, Tochigi, 329-0498 Japan; Department of Breast Oncology, Jichi Medical University School of Medicine, 3311-1 Yakushiji, Shimotsuke, Tochigi, 329-0498 Japan

**Keywords:** Trastuzumab, Anthracycline, Chemotherapy, Cardiotoxicity, Heart failure, Cardiac Troponin, Biomarker, Echocardiography

## Abstract

The humanized monoclonal antibody trastuzumab has been in routine use for chemotherapy for human epidermal growth factor receptor II (HER2)-positive breast cancer. A major adverse effect of trastuzumab is cardiotoxicity. Well-established biomarkers or echocardiographic parameters to predict trastuzumab-induced cardiotoxicity have not yet been determined. We attempted to identify useful biomarkers and/or echocardiographic parameters to predict trastuzumab-induced cardiotoxicity.

We prospectively investigated the cases of 19 women who received chemotherapy including anthracyclines and trastuzumab for HER2-positive breast cancer. We measured cardiac biomarkers and echocardiographic parameters before their chemotherapy and every 3 months up to 15 months until the end of the adjuvant trastuzumab therapy.

We divided the patients into two groups: group R was the nine patients who showed a reduction of left ventricular ejection fraction (LVEF) ≥5%, and group N was the 10 patients who showed a reduction of LVEF <5%. The high-sensitivity troponin T (hs-TnT) level at 6 months was significantly higher in group R than in group N (11.0 ± 7.8 pg/mL vs. 4.0 ± 1.4 pg/mL, *p* < 0.01). The hs-TnT level with a cutoff value of 5.5 pg/mL at 6 months had 78% sensitivity and 80% specificity for predicting a reduction of LVEF at 15 months. In our evaluation of echocardiographic parameters at baseline, the diastolic function was more impaired in group R than in group N.

The hs-TnT and echocardiographic parameters of diastolic function could be useful to predict trastuzumab-induced cardiotoxicity.

## Background

Trastuzumab is a humanized monoclonal antibody that has been in routine use for chemotherapy for human epidermal growth factor receptor II (HER2)-positive breast cancer. Several clinical trials revealed evidence that combination therapy with trastuzumab and anthracyclines improved the survival rate of patients with HER2-positive breast cancer, which is detected in 20% to 30% of all breast cancers and has both a poor prognosis and a high risk of recurrence (Hudis 2007). However, a known major adverse effect of trastuzumab is cardiotoxicity, which can cause the development of heart failure and necessitate the withdrawal of a therapeutic agent for breast cancer (Chen et al. 2008). The assessment of a reduction of the ventricular ejection fraction (LVEF) by echocardiography or cardiac scintigraphy has been used to detect trastuzumab induced-cardiotoxicity. It is an important issue to elucidate the markers to predict trastuzumab-induced cardiotoxicity before which show a reduction of LVEF.

Several reports showed that plasma concentration of high-sensitivity troponin I (hs-TnI) or a myocardial strain measured by echocardiography correlates with the risk of trastuzumab-induced cardiotoxicity, and thus the measurement of these parameters may be able to predict cardiotoxicity (Sawaya et al. 2011, 2012; Fallah-Rad et al. 2011). However, specific biomarkers or echocardiographic parameters to predict trastuzumab-induced cardiotoxicity remains to be established.

In this study, we prospectively investigated whether cardiac biomarkers and/or echocardiographic parameters predict the incidence of trastuzumab-induced cardiotoxicity, and we compared the operating characteristics of these parameters with those reported before.

## Methods

### Study design and patient selection

**Figure 1:**
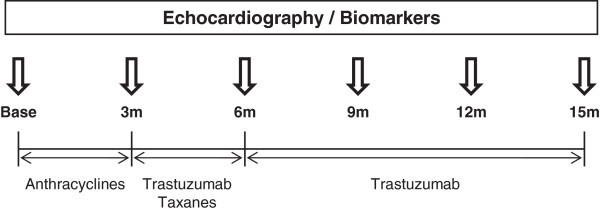
**Time course of the study protocol.** Base: baseline; m: months.

Twenty women with HER2-positive breast cancer and scheduled to receive adjuvant chemotherapy including anthracyclines, taxanes and trastuzumab at Jichi Medical University Hospital between June 2010 and March 2012 were prospectively enrolled. The patient population was evaluated before chemotherapy and every 3 months up to 15 months until the end of trastuzumab therapy: before the initiation of anthracycline therapy (at baseline), the completion of the anthracycline therapy (before the initiation of trastuzumab therapy: at 3 months), and at 6, 9, 12 and 15 months. At each time point, cardiac biomarkers and echocardiographic parameters were measured (Figure 1).

In accordance with the guideline of the Cardiac Review and Evaluation Committee for trastuzumab-associated cardiotoxicity, we defined trastuzumab-induced cardiotoxicity as a reduction of LVEF ≥5% to <55% with symptomatic heart failure or an asymptomatic reduction of LVEF ≥10% to <55%. Because none of the present 20 patients showed cardiotoxicity in accordance with this definition, we defined cardiac damage as a reduction of LVEF ≥5%, and we divided the patients into two groups: group R (reduction of LVEF ≥5%) and group N (reduction of LVEF <5%) for analysis. The Ethics Committee of Jichi Medical University approved the study protocol. All patients enrolled in this study provided informed consent.

### Measurement of biomarkers

The biomarkers assessed in this study were high-sensitivity troponin T (hs-TnT), high-sensitivity troponin I (hs-TnI), high-sensitivity C-reactive protein (hs-CRP), N-terminal pro-brain natriuretic peptide (NT-proBNP), serum creatinine (Cr), and the estimated glomerular filtration rate (eGFR). The levels of hs-TnT, hs-CRP and NT-proBNP were measured by an electrochemiluminescence immunoassay, latex-enhanced nephelometry, and an electrochemiluminescence sandwich immunoassay, respectively, according to the manufacturer’s instructions (Roche Diagnostics, Mannheim, Germany). The hs-TnT assay has an analytic range of 3–10,000 pg/mL, and the 99th percentile cutoff point has been reported as ≥14 pg/mL in healthy individuals (Giannitsis et al. 2010). The hs-TnI levels were measured using a chemiluminescence sandwich immunoassay according to the manufacturer’s instructions (Siemens Medical Solution Diagnostics, Tarrytown NY, USA). The hs-TnI assay has an analytic range of 6–50,000 pg/mL, and the 99th percentile cutoff point has been reported as ≥40 pg/mL in healthy individuals (Melanson et al. 2007). Cr was determined by a standard assay at Jichi Medical University Hospital, and the eGFR was calculated by the method defined by the Japan Association of Chronic Kidney Disease (eGFR = 194 × Cr^-1.094^ × age^-0.287^ × 0.739).

### Measurement of echocardiographic parameters

Transthoracic echocardiography was performed using the iE33 (Philips, Eindhoven, Netherlands) or ARTIDA (Toshiba Medical Systems Corp., Tochigi, Japan). All echocardiographic examinations were performed by experienced cardiologists (K.K. and M.I.) blinded to the biomarker results. Echocardiographic parameters were measured in accordance with the guidelines of the American Society of Echocardiography, and the following parameters were assessed: LVEF, left ventricular end-diastolic diameter (LVDd), mitral E-wave filling velocity/mitral A-wave filling velocity (E/A), deceleration time (DcT), peak early diastolic velocity of septal mitral annulus (e’).

LVEF was measured by a modified Simpson’s method except in one patient. Because this patient’s apical view was difficult to visualize due to expanders, the LVEF was measured by the M-mode method using the parasternal view, and the E/A, DcT, and e’ could not be evaluated.

### Statistical analysis

All data are expressed as the mean ± SD. Categorical variables are expressed as percentages and were analyzed using the χ-square test or Fisher’s exact test. The variables that were not normally distributed were logarithmically transformed before the analysis. The comparisons of variables between group N and group R at the same time points were done with Student’s *t*-tests. The comparison of variables within each group versus the baseline was performed with a repeated-measures analysis of variance (ANOVA) followed by Tukey’s test. Pearson’s correlation was used to test relationship between the changes of hs-TnT and LVEF. A receiver-operator characteristic (ROC) curve analysis was applied to determine the cutoff values, sensitivity and specificity for hs-TnT. A *p*-value <0.05 was considered significant. The software program SPSS (version 16.0, Chicago, IL) was used to perform the analysis.

## Results

Twenty women were prospectively enrolled in this study; one patient was excluded from the study because her chemotherapy protocol was changed because a malignant lymphoma developed during her breast cancer treatment. Therefore, 19 patients participated in and completed the study. They were divided into two groups: group R was nine patients and group N was 10 patients.

**Figure 2:**
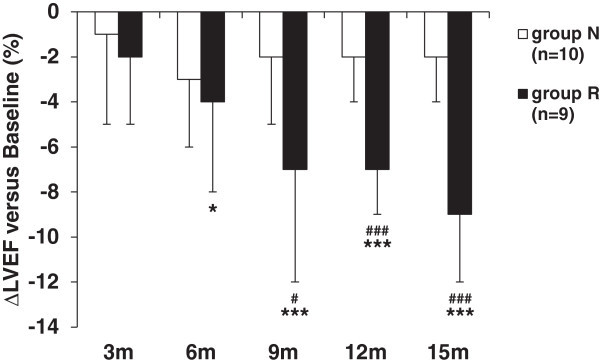
**The changes of left ventricular ejection fraction (ΔLVEF) at 3 months (3 m), 6 months (6 m), 9 months (9 m), 12 months (12 m) and 15 months (15 m) versus baseline.** Bars represent mean ± SD. ^#^ *p* < 0.05, ^##^ *p* < 0.01 and ^###^ *p* < 0.001 comparing group N vs. group R at the same time point. *p < 0.05, **p < 0.01 and ***p < 0.001 within each group vs. baseline with a repeated ANOVA followed by Tukey’s test.

The baseline characteristics are listed in Table 1. The body mass index values were significantly higher in group R than in group N (25 ± 3 vs. 22 ± 2, *p* < 0.05). There was no significant difference between the two groups in age, cardiovascular risk factors, side of breast cancer, radiation use, dose of anthracyclines, or renal function. In the evaluation of echocardiographic parameters at baseline, there was no significant difference in LVEF or LVDd between the two groups. The E/A and e’ values were significantly lower in group R than in group N (1.00 ± 0.36 vs. 1.44 ± 0.41, *p* < 0.05 and 7.6 ± 2.0 cm/s vs. 11.2 ± 3.2 cm/s, *p* < 0.05, respectively), and DcT was significantly longer in group R than in group N (227 ± 48 ms vs. 185 ± 26 ms, *p* < 0.05), showing that diastolic function was more impaired in group R than in group N.The changes of LVEF at 3, 6, 9, 12 and 15 months versus baseline are shown in Figure 2. At 9, 12 and 15 months, the LVEF was significantly reduced in group R compared to group N. In group R, the LVEF was significantly reduced at 6, 9, 12, and 15 months compared to baseline, whereas in group N, the LVEF was not changed at any time point. The reduction of LVEF at 15 months in group R was 9%.

**Table 1:** **Baseline characteristics of the 19 patients with HER2-positive breast cancer who showed normal (N) or reduced (R) left ventricular ejection fraction**

	Group N (n = 10)	Group R (n = 9)	***p-value***
Age (yrs)	49 ± 7	57 ± 9	0.071
Body mass index (kg/m2)	22 ± 2	25 ± 3	0.037
Cardiovascular risk factors			
Hypertension	1 (10%)	1 (11%)	0.941
Diabetes	0 (0%)	0 (0%)	
Hyperlipidemia	2 (20%)	2 (22%)	0.912
Smoking	2 (20%)	4 (44%)	0.277
Family history of CAD	1 (10%)	0 (0%)	0.357
Side of breast cancer			
Right	6 (60%)	8 (89%)	0.171
Left	4 (40%)	1 (11%)	
Bilateral	0 (0%)	0 (0%)	
Radiation	7 (70%)	5 (56%)	0.541
Chemotherapy			
Doxorubicin 240 mg/m2	1 (10%)	4 (44%)	0.098
Epirubicin 300 mg/m2	9 (90%)	5 (56%)	
Creatinine (mg/dL)	0.55 ± 0.10	0.50 ± 0.07	0.225
eGFR (mL/min/1.73 m2)	93.9 ± 19.0	100.5 ± 21.1	0.509
Echocardiographic parameters			
LVEF (%)	68 ± 5	71 ± 3	0.103
LVDd (mm)	44 ± 3	44 ± 4	0.756
E/A	1.44 ± 0.41	1.00 ± 0.36	0.028
DcT (ms)	185 ± 26	227 ± 48	0.040
e’ (cm/s)	11.2 ± 3.2	7.6 ± 2.0	0.019

CAD: coronary artery disease, eGFR: estimated glormerular filtration rate, LVEF: left ventricular ejection fraction, LVDd: left ventricular end-diastolic diameter, E/A: mitral E-wave filling velocity/mitral A-wave filling velocity, DcT: deceleration time, e’: peak early diastolic velocity of septal mitral annulus.

The changes in cardiac biomarkers are shown in Table 2. In group R, the hs-TnT levels were significantly elevated at 3 and 6 months compared to baseline. In group N, the hs-TnT levels were significantly elevated at 3 months compared to baseline. At 6 months, the hs-TnT levels in group R were significantly higher than those in group N (11.0 ± 7.8 pg/mL vs. 4.0 ± 1.4 pg/mL, *p* < 0.01).

**Table 2:** **Biomarker levels in the HER2-positive breast cancer patients who showed normal (N) or reduced (R) left ventricular ejection fraction**

Biomarkers	Group N (n = 10)	Group R (n = 9)	***p-value***
hs-TnT (pg/mL)			
Baseline	3.0	3.0	
3 months	7.0 ± 5.8**	9.2 ± 6.6*	0.524
6 months	4.0 ± 1.4	11.0 ± 7.8**	0.005
9 months	4.4 ± 2.7	3.6 ± 1.7	0.457
12 months	3.9 ± 1.6	4.9 ± 2.1	0.321
15 months	3.8 ± 1.0	4.4 ± 2.1	0.588
hs-TnI (pg/mL)			
Baseline	4.2 ± 4.0	2.8 ± 2.9	0.426
3 months	14.1 ± 7.0***	19.7 ± 17.3***	0.649
6 months	10.6 ± 6.7**	21.6 ± 16.4***	0.246
9 months	7.3 ± 4.8	7.3 ± 6.1	0.621
12 months	7.8 ± 5.9	8.7 ± 5.0 **	0.634
15 months	10.3 ± 3.5**	10.9 ± 6.4***	0.788
hs-CRP (mg/dL)			
Baseline	0.04 ± 0.02	0.14 ± 0.18	0.112
3 months	0.35 ± 0.27*	0.71 ± 0.64*	0.283
6 months	0.09 ± 0.09	0.11 ± 0.12	0.393
9 months	0.04 ± 0.03	0.05 ± 0.03	0.521
12 months	0.18 ± 0.42	0.08 ± 0.11	0.867
15 months	0.07 ± 0.07	0.85 ± 1.43	0.219
NT-proBNP (pg/mL)			
Baseline	66.0 ± 30.5	46.6 ± 43.5	0.071
3 months	56.4 ± 41.9	99.4 ± 76.9	0.337
6 months	45.3 ± 32.8	22.9 ± 13.5	0.112
9 months	39.5 ± 27.4	59.1 ± 27.2	0.093
12 months	59.9 ± 51.4	52.1 ± 28.0	0.886
15 months	61.1 ± 44.0	52.9 ± 26.4	0.795

*p < 0.05, **p < 0.01 and ***p < 0.001 vs. baseline within each group with a repeated ANOVA followed by Tukey’s test.

In group R, the hs-TnI levels were significantly elevated at 3, 6, 12 and 15 months compared to baseline. In group N, the hs-TnI levels were significantly elevated at 3, 6 and 15 months compared to baseline. There was no significant difference in hs-TnI levels between the two groups at any time point.

In both group R and group N, the hs-CRP levels were significantly elevated at 3 months compared to baseline. There was no significant difference in hs-CRP levels between the two groups at any time point.

In both group R and group N, the NT-proBNP levels were not significantly different from the baseline levels at any time point. There was no significant difference in NT-proBNP levels between the two groups at any time point.

**Figure 3:**
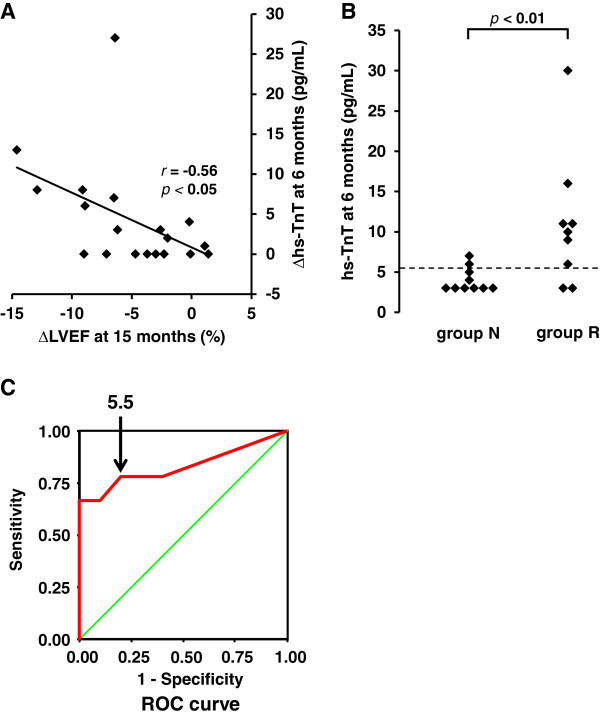
**The hs-TnT level at 6 months predicts a reduction of LVEF at 15 months.** **(A)** The correlation between the changes of high-sensitivity troponin T (∆hs-TnT) at 6 months and the changes of left ventricular ejection fraction (∆LVEF) at 15 months. **(B)** The dot diagram depicting the distribution of hs-TnT at 6 months in each group. **(C)** The ROC curve analysis of hs-TnT at 6 months in both groups. The transverse dot line in **(B)** and the arrow in **(C)** indicate the cutoff point of hs-TnT that provides the maximum value by adding sensitivity to specificity.

Figure 3A shows the changes of hs-TnT levels at 6 months significantly correlated with the changes of LVEF at 15 months (r = -0.56, *p* < 0.05). The distribution of hs-TnT levels at 6 months and the ROC curve analysis of hs-TnT levels at 6 months are shown in Figure 3B and Figure 3C, respectively. At 6 months, seven of the nine patients in group R were above the hs-TnT cutoff value of 5.5 pg/mL, providing 78% sensitivity and 80% specificity for predicting a reduction of LVEF at 15 months.

## Discussion

In this study’s search for predictors of cardiotoxicity in breast cancer treatment, two findings are notable. First, elevated hs-TnT levels at 6 months showed a possible ability to predict a subsequent reduction of LVEF. Second, the cardiac diastolic function at baseline was more impaired in group R than in group N.

Cardiac troponins are contractile regulatory peptides, and with cardiac muscle injury they spill into circulating blood. They are used as diagnostic biomarkers, especially for acute coronary syndrome (Donnelly and Millar-Craig 1998; Antman et al. 1996; Apple et al. 2005; Aviles et al. 2002; Lindahl et al. 2000). A high-sensitivity cardiac troponin assay that can detect low levels of circulating cardiac troponin has emerged, and its diagnostic and prognostic accuracy have been reported in several groups of patients with cardiovascular diseases such as subclinical cardiovascular diseases (deFilippi et al. 2010), heart failure (Latini et al. 2007), and stable coronary artery disease (Omland et al. 2009). This high-sensitivity troponin assay can measure two kinds of troponin, troponin T and I. There are several differences between troponin T and I; for example, troponin T has a larger molecular weight and a longer half-life in blood, and it is more affected by renal dysfunction than troponin I (Tsutamoto et al. 2009, 2010; Fehr et al. 2003). A recent report discussed the differences between the roles of troponin T and I in the prediction of cardiovascular events in stable coronary artery disease patients, and in that report, hs-TnI correlated moderately with hs-TnT (r = 0.44), and hs-TnI was associated with the incidence of myocardial infarction (Omland et al. 2013).

Our study is the first to reveal that in group R, both the hs-TnT levels and the hs-TnI levels not only at 3 months but also at 6 months were significantly higher than the corresponding values at baseline. Sawaya et al. (2012) reported the utility of hs-TnI to predict trastuzumab-induced cardiotoxicity. Their report showed that elevated hs-TnI levels at 3 months after the completion of anthracycline therapy could predict subsequent cardiotoxicity in breast cancer patients with adjuvant trastuzumab therapy, and that hs-TnI with a cutoff value of 30 pg/mL had 48% sensitivity and 73% specificity for detecting cardiotoxicity. However, when we look into the report in detail, Sawaya’s report showed that there were no significant differences in hs-TnI levels between the group with cardiotoxicity and the group without cardiotoxicity (32 pg/mL vs. 17 pg/mL, *p* = 0.18). In our study, the hs-TnT levels but not the hs-TnI levels at 6 months were significantly different between the two groups (11.0 ± 7.8 pg/mL vs. 4.0 ± 1.4 pg/mL, *p* < 0.01), and hs-TnT with a cutoff value of 5.5 pg/mL was predictive of a subsequent reduction of LVEF by nearly 10% at 15 months, suggesting that hs-TnT could be a more useful marker to predict cardiotoxicity than hs-TnI. The molecular size of troponin I is smaller than that of troponin T, which may facilitate transfer of troponin I spill into circulating blood, and induce a large variation in the hs-TnI levels of group N. This may indicate that the leakage of troponin T could more specifically reflect severe myocardial damage causing reduction of LVEF.

It is a novel finding that a continuous elevation of high-sensitivity troponins at 3 and 6 months, not only at 3 months, is correlated with the subsequent development of cardiotoxicity. Anthracycline therapy for breast cancer patients is generally completed at 3 months and then adjuvant trastuzumab therapy is initiated, and our results thus suggest that anthracycline-induced cardiotoxicity remains and trastuzumab-induced myocardial injury is added at 6 months. Trastuzumab is thought to inhibit a process of repairing myocardial injury caused by anthracyclines, leading to a subsequent reduction of LVEF. This hypothesis is in agreement with the previous report that the incidence of cardiotoxicity ranges from 2% to 7% when trastuzumab is used as a monotherapy, and up to 27% when trastuzumab is used with anthracyclines as adjuvant therapy (Yeh and Bickford 2009).

Onitilo et al. (2012) showed that elevated hs-CRP (≥0.3 mg/dL) during trastuzumab therapy had 93% sensitivity and 46% specificity for detecting cardiotoxicity, and that this value was useful especially for identifying patients at low risk of developing cardiotoxicity. Our study also showed that hs-CRP levels were significantly elevated at 3 months compared to baseline, although there were no significant differences between the two patient groups. This result is in agreement with Onitilo’s report and might reflect inflammations due to anthracycline-induced cardiotoxicity. On the other hand, in our study as well as in several previous reports (Sawaya et al. 2011, 2012; Fallah-Rad et al. 2011), the NT-proBNP levels of both group R and group N patients were not significantly different compared to those at baseline at any time points.

Our study is the first to show that the diastolic function at baseline was more impaired in group R than in group N. Several studies indicated the utility of decreased longitudinal strain (Sawaya et al. 2011, 2012; Fallah-Rad et al. 2011) and systolic velocity of septal mitral annulus (s’) (Fallah-Rad et al. 2011) measured by echocardiography for predicting the cardiotoxicity of adjuvant trastuzumab therapy. In our study, s’ was not significantly different at any time point because there was no patient with severe systolic dysfunction such as reduction of LVEF ≥10% to <55% (data not shown). However, we showed that at baseline, the E/A and e’ values were significantly lower and the DcT was significantly longer in group R than in group N, suggesting that echocardiographic parameters of diastolic function could be useful to identify patients at high risk of developing cardiotoxicity. These findings seem to be supported by some previous reports showing that diastolic dysfunction precedes or coexists with systolic dysfunction for various conditions, such as hypertensive heart disease and ischemic heart disease (Vasan and Levy 1996; Nishimura and Tajik 1997).

Our study has several limitations. First, we used a reduction of LVEF ≥5% as a surrogate marker of trastuzumab-induced cardiotoxicity. None of our patients developed heart failure, and the incidence of trastuzumab-induced cardiotoxicity was lower than in some other previous studies. The following points can be given as the reasons. In our patient population, the prevalence of underlying diseases such as diabetes, hypertension and obesity were lower than those in previous studies. These diseases are known to be conventional cardiac risk factors and have been reported as robust predictors of anthracycline-induced cardiotoxicity (Lotrionte et al. 2013). Another point was that the sensitivity for chemotherapy-induced cardiotoxicity could be different between races. In addition, myocardial strains measured by echocardiography were not assessed in our study. Moreover, our study was prospective but the population was small. Further studies with larger populations are needed to test the significance of hs-TnT in trastuzumab-induced cardiotoxicity.

## Conclusions

Our study clearly showed that the continuous elevation of hs-TnT at 3 and 6 months during adjuvant trastuzumab therapy could predict the subsequent reduction of LVEF, and diastolic function at baseline was more impaired in group R than in group N. In breast cancer patients treated with anthracyclines and trastuzumab, hs-TnT and echocardiographic parameters of diastolic function may be useful to predict cardiotoxicity, and they may be helpful as guides to avoid adverse cardiac effects.
